# *In Silico *identification of pathogenic strains of *Cronobacter *from Biochemical data reveals association of inositol fermentation with pathogenicity

**DOI:** 10.1186/1471-2180-11-204

**Published:** 2011-09-20

**Authors:** Stephen E Hamby, Susan Joseph, Stephen J Forsythe, Nadia Chuzhanova

**Affiliations:** 1 School of Science and Technology, Nottingham Trent University, Nottingham, NG11 8NS, UK

## Abstract

**Background:**

*Cronobacter*, formerly known as *Enterobacter sakazakii*, is a food-borne pathogen known to cause neonatal meningitis, septicaemia and death. Current diagnostic tests for identification of *Cronobacter *do not differentiate between species, necessitating time consuming 16S rDNA gene sequencing or multilocus sequence typing (MLST). The organism is ubiquitous, being found in the environment and in a wide range of foods, although there is variation in pathogenicity between *Cronobacter *isolates and between species. Therefore to be able to differentiate between the pathogenic and non-pathogenic strains is of interest to the food industry and regulators.

**Results:**

Here we report the use of Expectation Maximization clustering to categorise 98 strains of *Cronobacter *as pathogenic or non-pathogenic based on biochemical test results from standard diagnostic test kits. Pathogenicity of a strain was postulated on the basis of either pathogenic symptoms associated with strain source or corresponding MLST sequence types, allowing the clusters to be labelled as containing either pathogenic or non-pathogenic strains. The resulting clusters gave good differentiation of strains into pathogenic and non-pathogenic groups, corresponding well to isolate source and MLST sequence type. The results also revealed a potential association between pathogenicity and inositol fermentation. An investigation of the genomes of *Cronobacter sakazakii *and *C. turicensis *revealed the gene for inositol monophosphatase is associated with putative virulence factors in pathogenic strains of *Cronobacter*.

**Conclusions:**

We demonstrated a computational approach allowing existing diagnostic kits to be used to identify pathogenic strains of *Cronobacter*. The resulting clusters correlated well with MLST sequence types and revealed new information about the pathogenicity of *Cronobacter *species.

## Background

*Cronobacter*, formerly known as *Enterobacter sakazakii *[1], is a bacterial genus containing seven species [2,3] in the family *Enterobacteriacae; C. sakazakii, C. malonaticus, C. muytjensii, C. turicensis, C. dublinensis, C. universalis*, and *C. condimenti*. The organism has received a lot of attention recently due to its association with neonatal infections, especially meningitis, necrotizing enterocolitis, septicaemia and subsequent death [4,5]. These bacteria have been isolated from a wide range of food stuffs [6-8], therefore it is important to be able to detect *Cronobacter *species in food. For this purpose several diagnostic tests exist. However, most of these tests make no distinction as to the species of the bacteria. Not all *Cronobacter *species are known to be pathogenic to infants and can cause asymptomatic colonisation. The strict microbiological criteria for the presence of *Cronobacter *in powdered infant formula (< 1 *Cronobacter *cell/10 g) for intended age < 6 months [9] means it is of great interest to differentiate between pathogenic and non-pathogenic strains. Although a range of possible virulence features (i.e. ompA, adhesins, iron-uptake mechanisms) have been identified in *Cronobacter *and reviewed elsewhere [10], their presence does not correspond to clinical symptoms. Therefore, the identification of further discriminating factors would be useful. Currently, to differentiate between species, it is necessary to sequence either the 16S RNA subunit [11] or the MLST genes [12]; the latter is required for searching the *Cronobacter *MLST database [12,13]. There are 178 isolates of *Cronobacter *recorded in the MLST database [13] at the time of analysis (March 2011). Although it is known that type 4 strains (ST 4) are associated with meningitis [14], neither of the above methods is able to differentiate between pathogenic and non-pathogenic strains, they only identify individual species. Moreover, both methods are time consuming compared with the use of biochemical diagnostic test kits which take 4-18 hours to produce results that can easily be interpreted.

For this reason we aimed to develop methods for identifying which of the strains in the *Cronobacter *genus are pathogenic based on data obtained from standard biochemical diagnostic tests. These tests were those commonly used to identify *Cronobacter *isolates and are used in their taxonomic description [2,3,11]. Here we used Expectation Maximization (EM) clustering algorithm to divide the data on the basis of the biochemical test results. Since the precise pathogenic status of most *Cronobacter *strains is unknown, we considered the resulting clusters as being pathogenic or not on the basis of (a) the source from which the strains were isolated and/or (b) MLST types previously associated with pathogenic or non-pathogenic strains (see Materials and Methods) and reference [14]. The clustering of the biochemical test results was also examined for traits associated with pathogenicity.

## Results and Discussion

Clustering the dataset for Test 1 with the number of clusters being 2, resulted in clusters 1 (*p_1 _*= 0.26) and 2 (*p_2 _*= 0.74) containing 25 and 65 strains respectively (*L *= -3.119; Table 1) where *p_i _*(*i *= 1, 2) is the probability of cluster membership for a randomly chosen strain and *L *is the maximum log likelihood (see Materials and Methods). According to our hypothesis cluster 2 was most likely to contain pathogenic strains since all ST 4 strains were assigned to this cluster. It is known that ST 4 strains are associated with the most serious pathogenic states such as meningitis in infants [14]. Of the other MLST types, ST 1 and 3 were placed exclusively with the potentially non-pathogenic strains in cluster 1. ST 7 was split between two clusters with 7 of 11 strains in the non-pathogenic grouping. All except one ST 8 strain were predicted to be in the pathogenic cluster, as were all of the ST 12 strains (Table 1). The group with unspecified clinical source (22 strains) was divided between the two clusters, indicating that not all clinical isolates are likely to be pathogenic and this feature (isolation of a strain from a clinical sample) alone by no means allows us to infer pathogenicity of a strain. For example, one clinical case, classified as non-pathogenic, was obtained from a breast abscess and it is plausible that this was a secondary infection although it is not known if another infectious agent was isolated. Thus this may indeed be a non-pathogenic strain. Two asymptomatic strains appeared in the pathogenic cluster; one of these strains is ST 12 and the other ST 13. Several ST 12 strains are from clinical sources and it is likely that all ST 12 strains will have similar pathogenic characteristics. Therefore, we can speculate that these strains could have caused an infection following a higher ingested dose or a lower immune status.

**Table 1:** Clusters from Test 1 dataset

*Cronobacter *species	MLST type	Cluster 1: potential non-pathogenic Source (number of strains)	Cluster 2: potential pathogenic Source (number of strains)
*C. sakazakii*	1	IF(4), C(1), MP(1), Faeces(1)	IF(1)

*C. sakazakii*	3	IF(1), EFT(2), FuF(4), U(1)	

*C. sakazakii*	4		C(9), IF(7), MP(1), Washing Brush(1), E(1), U(2)

*C. sakazakii*	8	C(1)	C(6), IF(1)

*C. sakazakii*	12		C(3), U(1)

*C. sakazakii*	13		IF(1), C(1)

*C. sakazakii*	15		C(1)

*C. sakazakii*	16		Spices(1)

*C. sakazakii*	17		IF(1)

*C. sakazakii*	18		C(1)

*C. sakazakii*	21		F(1)

*C. sakazakii*	31		C(1)

*C. sakazakii*	35		Herbs(1)

*C. sakazakii*	40		F(1)

*C. sakazakii*	41		C(1)

*C. malonaticus*	7	C(5), F(1), Faeces(1)	C(2), MP(1), WF(1)

*C. malonaticus*	10		Herbs(2)

*C. malonaticus*	11	C(1)	C(2)

*C. malonaticus*	29		U(1)

*C. turicensis*	5		MP(1), Herbs(1), MP(1), C(2)

*C. turicensis*	19		U(1)

*C. turicensis*	32		IF(1)

*C. turicensis*	37		Herbs(1)

*C. muytjensii*	33		U(1)

*C. muytjensii*	34		U(1)

*C. dublinensis*	42		U(1)

*C. dublinensis*	43		U(1)

*C. universalis*	54		Freshwater(1)

Abbreviations: C: clinical, E: Environmental, EFT: Enteral Feeding Tube, F: Food, FuF: Follow up Formula, IF: Infant Formula, MP: Milk Powder, U: Unknown WF: Weaning Food. Sources of isolation and strain numbers are given in full in Additional File 1.

Clustering for the Test 2 dataset gave two clusters in which 84 strains (91% of the data) were in cluster 2 (*p_2 _*= 0.9) and eight strains (9% of the data) were in cluster 1 (*p_1 _*= 0.1, *L *= -6.44; Table 2). One strain of those in cluster 1 was associated with a clinical diagnosis (ST 31) and was likely to be pathogenic, as well as one ST 4 strain, with the remainder placed in cluster 2. The heterogeneity of MLST types in both clusters, as well as the small number of strains in cluster 1, suggests that the biochemical data in Test 2 is not sufficient to differentiate between pathogenic and non-pathogenic strains. To prove this, the EM algorithm was allowed to automatically determine the number of clusters to assign the data to (data not shown). As a result, only a single cluster was produced indicating that the Test 2 data is not sufficient to differentiate between *Cronobacter *strains.

**Table 2:** Clusters from Test 2 dataset

*Cronobacter *species	MLST Type	Cluster 1: potential non-pathogenic Source (number of strains)	Cluster 2: potential pathogenic Source (number of strains)
*C. sakazakii*	1	IF(1)	IF(4), C(1), MP(1), Faeces(1)

*C. sakazakii*	3		IF(1), FuF(4), WF(1), U(1)

*C. sakazakii*	4	IF(1)	C(9), IF(6), MP(1), WF(1), E(1), Washing Brush(1), U(2)

*C. sakazakii*	8		C(7), IF(1)

*C. sakazakii*	9		WF(1)

*C. sakazakii*	12	C(1)	C(2), WF(1), U(2)

*C. sakazakii*	13		C(1), IF(1)

*C. sakazakii*	15		C(1)

*C. sakazakii*	16		Spices(1)

*C. sakazakii*	17		IF(1)

*C. sakazakii*	18		C(1)

*C. sakazakii*	21		F(1)

*C. sakazakii*	31	C(1)	

*C. sakazakii*	40		F(1)

*C. sakazakii*	41		C(1)

*C. malonaticus*	7	C(1)	C(6), F(1), MP(1), WF(1), Faeces(1)

*C. malonaticus*	10		Herbs(2)

*C. malonaticus*	11	C(1)	C(2)

*C. malonaticus*	29		U(1)

*C. muytjensii*	33		U(1)

*C. muytjensii*	34	U(1)	

*C. turicensis*	37		Herbs(1)

*C. turicensis*	5		MP(1), Herbs(1), C(2)

*C. turicensis*	19		U(1)

*C. turicensis*	32		IF(1)

*C. turicensis*	35		Herbs(1)

*C. dublinensis*	36		U(1)

*C. dublinensis*	42	U(1)	

*C. dublinensis*	43		U(1)

*C. universalis*	54		Freshwater(1)

For abbreviations in this table see footnote to Table 1. Sources of isolation and strain numbers are given in full in Additional File 1.

Clustering of the Test 3 dataset (Table 3) resulted in cluster 1 containing 40 instances (*p_1 _*= 0.61) and cluster 2 containing 25 instances (*p_2 _*= 0.39, *L *= -16.726). The majority of the ST 4 strains were grouped in the second cluster, indicating that this cluster contains the potentially pathogenic strains. However, all other MLST types (with multiple strains available) were split between the two clusters. ST 1 was mostly placed in the non-pathogenic cluster, with one strain in cluster 2. ST 3 was split evenly (three in each) between the two clusters. Most of the ST 7 strains were found to be non-pathogenic with just one strain being pathogenic. However, many strains indicated as pathogenic in the Test 1 results (and also Test 2) were placed in the larger potentially non-pathogenic grouping. Based on the division of strains of the same MLST type between clusters, it is likely that the results of Test 3 are less accurate than Test 1 and Test 4 (see below), although many ST 1 and ST 4 strains appeared to be correctly assigned. Note that this test has the fewest number of strains available; it is expected that the availability of more data will greatly improve the results of clustering using this diagnostic test data.

**Table 3:** Clusters from Test 3 datasets

*Cronobacter *species	MLST Type	Cluster 1: potential non-pathogenic Source (number of strains)	Cluster 2: potential pathogenic Source (number of strains)
*C. sakazakii*	1	IF(4), C(1), Faeces(1)	MP(1)

*C. sakazakii*	3	IF(1), FuF(2)	FuF(2), U(1)

*C. sakazakii*	4	C(5), IF(1), Washing Brush(1)	C(3), IF(6), MP(1), E(1), U(1)

*C. sakazakii*	8	C(3)	C(2)

*C. sakazakii*	9	WF(1)	

*C. sakazakii*	12	U(1), WF(1)	C(1)

*C. sakazakii*	13	C(1)	

*C. sakazakii*	14	IF(1)	

*C. sakazakii*	15	C(1)	

*C. sakazakii*	16	Spices(150)	

*C. sakazakii*	17	IF(1)	

*C. sakazakii*	18	C(1)	

*C. sakazakii*	21	F(1)	

*C. sakazakii*	31		C(1)

*C. malonaticus*	7	C(2), WF(1), Faeces(1)	C(1)

*C. malonaticus*	10	Herbs(1)	

*C. malonaticus*	11		C(1)

*C. turicensis*	5	C(1)	MP(1) C(1)

*C. turicensis*	19	U(1)	

*C. turicensis*	32	Infant Food(1)	

*C. dublinensis*	36	U(1)	

*C. dublinensis*	38	U(1)	

*C. dublinensis*	42	U(1)	

*C. universalis*	54		Freshwater(1)

For abbreviations in this table see footnote to Table 1. Sources of isolation and strain numbers are given in full in Additional File 1.

For the fourth test, cluster 1 contained 33 strains (*p_1 _*= 0.44) and cluster 2 contained 43 strains (*p_2 _*= 0.56). The clusters are shown in Table 4 (*L *= -2.598). This clustering assignment was successful at differentiating between MLST types. ST 1 and 3 were placed entirely in the non-pathogenic grouping (cluster 1) and with two exceptions (strains 552, 553), the ST 4 strains were placed in cluster 2, allowing us to label the latter as the potentially pathogenic cluster. All except two ST 7 strains (strains 515, 535) were placed in the non-pathogenic cluster. ST 8 strains were placed in the pathogenic cluster as were all except one strain of ST 12 (strain 520). A more detailed examination of the strains allocated to each cluster showed that all strains labelled as pathogenic were positive for the inositol fermentation (Ino) test, whilst the prospective non-pathogenic strains were negative for this test. Although this is not conclusively shown by the result of the Inositol test in Test 1 and Test 2, the Test 1 data does indicate a bias towards strains with inositol fermentation in the pathogenic cluster. This suggested that either inositol fermentation was a requirement for pathogenicity, or that the genetic locus conferring inositol fermentation was linked to genes conferring pathogenic traits. This latter conclusion was supported by the two apparently pathogenic ST 4 strains which were negative for inositol fermentation (strains 552 and 553): strain 552 was isolated from infant formula, but strain 553 was associated with neonatal meningitis indicating pathogenesis. It is probable that the inositol fermentation gene was lost from these strains, but the pathogenic traits acquired alongside it remained. It should be noted that this test is different from the INO test in the Test 2 dataset, which we removed from the analysis as it produces the same result for all *Cronobacter *strains.

**Table 4:** Clusters from Test 4 dataset

*Cronobacter *species	MLST Type	Cluster 1: potential non-pathogenic Source(number of strains)	Cluster 2: potential pathogenic Source (number of strains)
*C. sakazakii*	1	IF(5), C(1), Faeces(1)	

*C. sakazakii*	3	IF(1), EFT(2), FuF(4), WF(1), U(1)	

*C. sakazakii*	4	C(1), IF(1)	C(8), IF(6), MP(1), WF(1), E(1), Washing Brush(1), U(2)

*C. sakazakii*	8		C(7), IF(1)

*C. sakazakii*	9	WF(1)	

*C. sakazakii*	12	C(1)	C(2), WF(1), U(1)

*C. sakazakii*	13		IF(1), C(1)

*C. sakazakii*	14	IF(1)	

*C. sakazakii*	15		C(1)

*C. sakazakii*	16		Spices(1)

*C. sakazakii*	17		IF(1)

*C. sakazakii*	18		C(1)

*C. malonaticus*	7	C(6), F(1), WF(1), Faeces(1)	C(1), MP(1)

*C. malonaticus*	10		Herbs(2)

*C. malonaticus*	11	C(2)	C(1)

All strains in cluster 1 (non-pathogenic) are negative for inositol fermentation, all strains in cluster 2 are positive for inositol fermentation. For abbreviations in this table see footnote to Table 1. Sources of isolation and strain numbers are given in full in Additional File 1.

### Consensus Clustering

Aggregating the clustering assignments based on the majority rule (two out of four) for the 48 strains which have data available from all four tests resulted in the clusters shown in Table 5. The results showed the majority of ST 4 strains were placed in cluster 2. However, there was still splitting of ST 1, 3 and 7 strains between the two clusters. There were also only 10 of the 48 strains placed in the non-pathogenic category. It was hypothesised that the results from Test 2 could be skewing the results, as this test did not differentiate between strains of different MLST sequence types. Therefore we excluded this test from the consensus clustering allowing 50 strains for which data was available from Tests 1, 3 and 4 to be analysed. Test 3 was retained since many ST 1 and ST 4 strains appeared to be correctly assigned. The results (Table 6) were similar to those for clustering with Test 4 alone. All strains of ST 1, 3 and 7 appeared in cluster 1 (the potential non-pathogenic grouping). With two exceptions (strains 552, 553), the ST 4 strains were grouped in cluster 2 (potentially pathogenic strains) along with the remainder of MLST types. The consensus clustering of Tests 1, 3 and 4 datasets also showed the same correlation with inositol fermentation as the results for Test 4 alone.

**Table 5:** Consensus clustering generated from Tests 1-4 data

*Cronobacter *species	MLST Type	Cluster 1 potential non-pathogenic: Source(number of strains)	Cluster 2 potential pathogenic: Source (number of strains)
*C. sakazakii*	1	IF(3), C(1), Faeces(1)	IF(1), MP(1)

*C. sakazakii*	3	IF(1), FuF(2)	FuF(2), U(1)

*C. sakazakii*	4		IF(7), C(6), MP(1), E(1), U(1), Washing Brush(1)

*C. sakazakii*	8		C(5)

*C. sakazakii*	12		U(1)

*C. sakazakii*	13		C(1)

*C. sakazakii*	15		C(1)

*C. sakazakii*	16		C(1)

*C. sakazakii*	17		IF(1)

*C. sakazakii*	18		C(1)

*C. malonaticus*	7	C(1), Faeces(1)	C(2), WF(1)

*C. malonaticus*	10		Herbs(1)

*C. malonaticus*	11		C(1)

All strains in cluster 1 (non-pathogenic) are negative for inositol fermentation, all strains in cluster 2 are positive for inositol fermentation. For abbreviations in this table see footnote to Table 1. Sources of isolation and strain numbers are given in full in Additional File 1.

**Table 6:** Consensus clustering generated from Tests 1, 3 and 4 data

*Cronobacter *species	MLST Type	Cluster 1: potential non-pathogenic Source (number of strains)	Cluster 2: potential pathogenic Source (number of strains)
*C. sakazakii*	1	IF(4), C(1), MP(1), Faeces(1)	

*C. sakazakii*	3	IF(1), FuF(4), U(1)	

*C. sakazakii*	4	C(1), IF(1)	C(7), IF(5), MP(1), E(1), Washing Brush(1), U(1)

*C. sakazakii*	8		C(5)

*C. sakazakii*	12		U(1)

*C. sakazakii*	13		C(1)

*C. sakazakii*	15		C(1)

*C. sakazakii*	16		Spices(1)

*C. sakazakii*	17		IF(1)

*C. sakazakii*	18		C(1)

*C. malonaticus*	7	C(3), Faeces(1), WF(1)	

*C. malonaticus*	10		Herbs(1)

*C. malonaticus*	11		C(1)

All strains in cluster 1 (non-pathogenic) are negative for inositol fermentation, all strains in cluster 2 are positive for inositol fermentation. For abbreviations in this table see footnote to Table 1. Sources of isolation and strain numbers are given in full in Additional File 1.

The results of all four clustering analyses gave plausible assignments of the data into two clusters, one of which has the propensity of being pathogenic and the other one of being non-pathogenic. The various MLST types were not divided equally between the clusters as one would expect by chance alone. Our hypothesis that strains with similar biochemical characteristics will have similar pathogenicity seems to hold since strains with pathogenic source isolates were grouped together throughout, although there were a small number of strains that were placed in the non-pathogenic cluster whilst having putative pathogenic status. Strains with the same MLST type were generally grouped together indicating, as might be expected, that strains with the same MLST type have similar biochemical characteristics.

To further investigate the association of inositol fermentation with pathogenicity, we examined the annotated genome of *C. sakazakii *BAA-894 [Genbank: CP000783] (strain 658) [15] for genes associated with inositol fermentation. Whilst BAA-894 is ST 1 and negative for inositol fermentation, this strain was isolated from powdered formula associated with a clinical outbreak [15] and therefore is likely to be a pathogenic strain. The gene coding for inositol monophosphatase [Genbank: ESA_00718, EC:3.1.3.25], which is annotated in the KEGG database [16] as part of the inositol phosphate metabolism pathway [KEGG: esa00562], was found in close proximity (approx 41 kb upstream) to a predicted protein [Genbank: ESA_00756] which has been identified in the BAA-894 genome and found in two other meningitic strains of *C. sakazakii *(strains 701, 767) by hybridization with the BAA-894 genome [15]. Strains 701 and 767 are ST 4 and were associated with fatal outbreaks, indicating this as a putative virulence factor. This was also found to be in close proximity to the zinc-containing metalloprotease locus characterized by Kothary et al [17]. Also at a distance of approximately 82 kb upstream, was a prophage fragment, GR3 [Genbank:ESA_00604-ESA_00630], which contains genes homologous to the *Yersinia pseudotuberculosis *adhesion pathogenicity island, as well as genes identified in strains 701 and 767 and the reference genome [Genbank: BAA-894]. Despite BAA-894 being deficient for inositol fermentation, the proximity of these genes to inositol monophosphatase and their implication as putative virulence factors suggests that the inositol monophosphate gene is associated with pathogenesis and supports our hypothesis that inositol fermentation is linked to the pathogenicity of *Cronobacter *species. The lack of inositol fermentation in BAA-894 may be explained by the loss of another gene, as yet unknown, which also plays a crucial role in the inositol phosphate metabolism pathway.

The genome of a *C. turicensis *strain [Genbank: FN543093-FN543096, ST 19, strain 1211] has also been sequenced [18]. No biotyping data exists for *C. turicensis *strains. However, the original characterisation of the *C. turicensis *species [2] showed that *C. turicensis *is positive for inositol fermentation and the *C. turicensis *strain sequenced contains the inositol monophosphatase gene associated with pathogenesis. The majority of *C*. *turicensis *strains were placed in the pathogenic cluster in Tests 1 and 2, but not in Test 3 (no data on *C. turicensis *is available for Test 4). The sequenced strain 1211 was pathogenic in Tests 1 and 2 (Tables 1 and 2).

Our clustering method has demonstrated that it is possible to quickly differentiate between pathogenic and non-pathogenic strains, and may lead to a quick and easy diagnostic test that can reliably identify pathogenic strains.

## Conclusions

Here we have used Expectation Maximization clustering to divide strains of *Cronobacter *into groups of pathogenic and non-pathogenic strains based on the results of diagnostic biochemical tests. The clustering assignments showed promise, clearly dividing the data into two clusters containing obviously pathogenic and non-pathogenic strains, based on the source of isolate and the MLST type of the strain. However, further experiments characterising the pathogenicity of *Cronobacter *strains are required to confirm the accuracy of the classification. Nevertheless, our results demonstrated a clear association between pathogenic strains and inositol fermentation, supported by genomic proximity of putative virulence factors to the gene coding for inositol monophosphatase.

## Methods

### Sources of bacterial strains

A total of 98 *Cronobacter *strains were analyzed in this study. Strains were from diverse food, clinical and environmental sources worldwide. The following species of *Cronobacter *were included: *C. sakazakii *NCTC 11467^T^, *C. malonaticus *LMG 23826^T^, *C. turicensis *LMG 23827^T^, *C. muytjensii *ATCC 51329^T^, *C. dublinensis *LMG 23823^T^, *C. universalis *NCTC 9529^T^. Strains were kindly donated by the following organizations: Health Products and Food Branch (Health Canada); CDC(Atlanta, USA); Children's Hospital (Los Angeles CA, USA); Northern Foods (UK); Oxoid ThermoFisher Ltd. (Basingstoke, UK); Hospital Cèské Budéjovice (Czech Republic); Institut fûr Tierärztliche Nahrungsmittelkunde Milchwissenschaften (Justus-Liebig-Universität Gießen, Germany); Nottingham City Hospital Trust (Nottingham, UK) and the Department of Medical Microbiology, Radboud (Nijmegen, Netherlands). All other strains were food and environmental isolates from the culture collection at Nottingham Trent University (Nottingham, UK) [19].

### Dataset

We examined results from four sets of diagnostic tests carried out on a total of 98 strains encompassing six species of *Cronobacter*. For a complete list of strains used in this work and their details see Additional File 1 and references [[1-3,15,18] and [20-28]]. Each test comprises a series of enzyme assays which produce a colour change recorded by the user. Bacterial species can then be identified by a characteristic series of changes in colour. All tests were carried out in accordance with the manufacturers' instructions and replicated three times; biotyping was performed as in [1]. The tests were those commonly used in the identification of *Cronobacter *species, and in taxonomic descriptions of the genus [2,3,12,19].

The four tests were:

#### Test 1

API 20 E (bioMérieux; SA, Marcy-l'Etoile, France) [29] consists of 20 enzyme assays scored as positive or negative. The assays are in the form of a strip of 20 cupules each containing a dehydrated substrate to which the reagents are added, for details of the specific tests see [29] and the manufacturers' instructions. Gram negative bacterial species are identified by comparison to an online database.

#### Test 2

ID 32E (bioMérieux SA; Marcy-l'Etoile, France) [30] consists of 32 miniaturised enzyme assays with positive or negative scores these assays can be measured either manually or automatically and Gram negative bacterial species are identified by comparison to an online database.

#### Test 3

API Zym (bioMérieux SA; Marcy-l'Etoile, France) [31] consists of 20 cupules with 19 enzyme assays and one control. The assays produce a coloured response which is scored in intensity between 0 and 5.

#### Test 4

Biotyping [1] is a series of biochemical tests for identifying bacteria. Tests are carried out for: indole production (Ind), motility at 36°C (Mot), acid production from *i*-inositol (Ino), malonate utilization (Malo) ornithine-Moellers (Orn), acid production from dulcitol (Dul), Methyl Red test (MR), Voges-Proskauer (VP) test, gas production (Gas), and nitrite metabolism (Nit). Details of all tests are given in [1].

The results of each test were represented by a separate dataset containing only the strains that have results for that test. The Test 1, Test 2, Test 3 and Test 4 datasets contained 91, 92, 65 and 76 strains respectively. There are 98 strains in total, 48 of these have data for all four tests. Further, 31 only have data for three out of four tests, and 14 for only two out of four tests. It should be noted that although there was a considerable overlap between the datasets, each dataset was considered separately. Each strain was identified by its isolate number retrieved from the *Cronobacter *MLST database [13] as well as source, geographical location and date of isolation. These attributes were removed for the purpose of clustering but were used to label the data afterwards. The result of each enzyme assay was represented categorically. In the case of Tests 1, 2 and 4 this was 0 or 1 for a negative or positive result respectively. A positive result being one which shows activity for the enzyme in the sample. Test 3 had categories ranging from 0 to 5. 0 is indicative of no reaction, and categories 1-5 indicate a range of positive responses, with 5 being the strongest response. Thus, each strain from each dataset was represented by a vector of attributes with each attribute containing the result of one of the enzyme assays in the corresponding test.

### Features used

The enzyme assays used in this study were not designed to discriminate between species or genotypes of *Cronobacter*. In all four tests there were assays where all (or almost all) strains were reported as producing the same result, either positive or negative. Attributes where all strains produce the same result, either positive or negative, for Tests 1, 2 and 4 or where all strains occupy one category in the case of Test 3 were removed from the list of features used for clustering. The features from each test used to perform clustering are listed in Table 7.

**Table 7:** Features used for clustering in each set of biochemical tests

Attributes Used	Attributes Removed
Test 1: LDC, ODC, CIT, URE, TDA, IND, VP, GEL, MAN, INO, SOR, RHA, SAC	Test1: ONPG, H2S, GLU, MEL, AMY, ARA, OX,

Test2: GAL, ACT, SAC, NAG, LAT, ARA, CEL, RAF, MAL, TRE, 2KG, MDG, SOR, XYL, RIB, GLY, RHA, PLE, ERY, MEL, GRT, MLZ, GNT, LVT, MAN, LAC, GLU, SBE, GLN, ESC	Test2: INO

Test3: Alkaline Phosphatase, Esterase, Esterase Lipase, Lipase, Leucine arylamidase, Valine arylamidase, Cystine arylamidase, Trypsin, α-chymotrypsin, Acid phosphatise, Naphthhol-AS-BI-phosphohydrolase, α-galactosidase, β-galactosidase, β-glucuronidase, α-glucosidase, β-glucosidase, N-acetyl- β-glucosaminidase, α-mannosidase, α-fucosidase	Test3: None

Test4:VP, MR, Nit, Orn, Mot, Ino, Malo, Gas	Test4: Dul, Ind

### EM Clustering

Clustering was carried out using the Expectation Maximization (EM) algorithm [32] implemented in the Weka machine learning package [33]. Whilst we give a brief explanation of the algorithm here, the reader is advised to consult the reference for full details. The algorithm operates by using Gaussian mixture models to estimating the maximum likelihood of membership in a cluster. In Gaussian mixture models the data is drawn from a mixture of *k *Gaussian distributions with mean *u_i _*and standard deviation *σ_i _*(1≤*i*≤*k*). The algorithm begins by randomly selecting parameters *u_i _*and *σ_i _*and computing the probability of cluster membership for each data point based on the probability density function defined by parameters *u_i _*and *σ_i_*. The distribution parameters are then re-estimated, the cluster membership is recomputed and these steps are repeated until a termination threshold is reached and/or the procedure converges to a local maximum of the likelihood function. For a two-component mixture model used in this study the resulting probabilities of a random strain being in class 1 and 2 were denoted by *p_1 _*and *p_2 _*(*p_1_*+*p_2 _*= 1) respectively, the maximum of log-likelihood estimate was denoted by *L*.

The following initial parameters for the EM algorithm were used: the maximum number of iterations was set to 100, the minimum standard deviation was set to 1.0E-06, and the number of clusters was set to 2.

The number of clusters was pre-specified for all experiments in this work; we set the number of clusters to two as we were seeking to split the data into pathogenic and non-pathogenic groups. Evaluation of the pathogenicity of the resulting clusters was somewhat subjective since the pathogenic status of the majority of *Cronobacter *strains was not known. However, some samples were clearly the source of pathogenic effects such as meningitis or septicaemia. There was evidence that strains with MLST sequence type (ST) 4 cause the most severe infections [14]. This was supported by the fact that all except two of ten strains that demonstrated clinical diagnosis were ST 4. Since strains with similar biochemical properties are likely to have similar pathogenic status, it was hypothesised that if the majority of ST 4 strains are placed into one cluster then this cluster is likely to be pathogenic whereas the remaining cluster is likely to be non-pathogenic. Therefore we designated the cluster with the largest number of ST 4 strains as pathogenic.

Since it is reasonable to assume that similar MLST types will have similar levels of pathogenicity, the spectrum of MLST types in each cluster is a good indicator of the accuracy of the assignment, and takes into account factors such as differences between species of *Cronobacter*. To date only a few plausible virulence features have been identified, such as ompA, adhesins, and iron-uptake mechanisms, many of which are distributed across the seven *Cronobacter *species [10].

### Consensus clustering

Consensus clustering was carried out to combine the results generated by the four tests. It was hypothesised that the consensus clustering will result in a more accurate classification of strains in the appropriate cluster. The four clustering assignments were combined by way of each assignment having one vote with the majority determining the cluster assignment of each strain. Any tie (i.e. two of four votes for each cluster) in the voting resulted in the strain being placed in the pathogenic cluster; this decreased the probability of missing a pathogenic strain while increasing the risk of finding a false positive. However, this was accepted as a good compromise, since missing a pathogenic strain has more serious consequences than misidentifying a negative strain. The consensus clustering was carried out on the 48 strains for which data for all four diagnostic tests is available.

## Abbreviations

C: Clinical; E: Environmental; EFT: Enteral Feeding Tube; EM: Expectation Maximization; F: Food; FuF: Follow up Formula; IF: Infant Formula; MLST: Multi Locus Sequence Typing; MP: Milk Powder; ST: Sequence Type; U: Unknown; WF: Weaning Food; For abbreviations of chemical tests, please see the appropriate sections of the text and references.

## Authors' contributions

SH conducted all computational analysis and drafted the manuscript, SJ acquired the experimental data and assisted writing and editing the manuscript, SJF and NC conceived and coordinated the study and helped to draft and edit the manuscript. All authors read and approved the final manuscript.

## Supplementary Material

Additional File 1 ***Cronobacter *strains**. Strains used in this study including source of isolation, MLST Type, references and which experiments they were used in. Click here for file

## References

[B1] Farmer JJ Asbury MA Hickman FW Brenner DJ The Enterobacteriaceae study group *Enterobacter sakazakii*: a new species of "*Enterobacteriaceae*" isolated from clinical specimens Intl J System Bacteriol 1980 30 569 584 10.1099/00207713-30-3-569

[B2] Iversen C Mullane N McCardell B Tall B Lehner A Fanning S Stephan R Joosten H *Cronobacter *gen. nov., a new genus to accommodate the biogroups of *Enterobacter sakazakii*, and proposal of *Cronobacter sakazakii *gen. nov., comb. nov., *Cronobacter malonaticus *sp. nov., *Cronobacter turicensis *sp. nov., *Cronobacter muytjensii *sp. nov., *Cronobacter dublinensis *sp. nov., *Cronobacter *genomospecies 1, and of three subspecies, *Cronobacter dublinensis *subsp. *dublinensis *subsp. nov., *Cronobacter dublinensis *subsp. *lausannensis *subsp. nov. and *Cronobacter dublinensis *subsp. *lactaridi *subsp. nov Intl J System Evol Microbiol 2008 58 1442 1447 10.1099/ijs.0.65577-0 18523192

[B3] Joseph S Cetinkaya E Drahovska H Levican A Figueras M Forsythe SJ *Cronobacter condimenti sp. nov.*, isolated from spiced meat and *Cronobacter universalis sp. nov.*, a novel species designation for *Cronobacter *sp. genomospecies 1, recovered from a leg infection, water and food ingredients Intl J System Evol Microbiol 2011 in press ecopy available 10.1099/ijs.0.032292-0 22661070

[B4] Food and Agriculture Organization-World Health Organization Joint FAO/WHO workshop on *Enterobacter sakazakii *and other microorganisms in powdered infant formula, Geneva, 2-5 February, 2004 2004

[B5] Food and Agriculture Organization-World Health Organization *Enterobacter sakazakii* and *Salmonella *in powdered infant formula 2006 Second Risk Assessment Workshop 16-20th January WHO Rome, Italy

[B6] Forsythe S *Enterobacter sakazakii *and other bacteria in powdered infant milk formula Maternal Child Nutr 2005 1 51 58 10.1111/j.1740-8709.2004.00002.x PMC6874386 16881878

[B7] Iversen C Forsythe SJ Risk profile of *Enterobacter sakazakii*, an emergent pathogen associated with infant milk formula Trends in Food Sci Technol 2003 11 443 454

[B8] Friedemann M *Enterobacter sakazakii *in food and beverages (other than infant formula and milk powder) Intl J Food Microbiol 2007 116 1 10 10.1016/j.ijfoodmicro.2006.12.018 17331606

[B9] Codex Alimentarius Commission Code of hygienic practice for powdered formulae for infants and young children ALINORM 08/31/13

[B10] Kucerova E Joseph S Forsythe SJ The *Cronobacter *genus: ubiquity and diversity Quality Assurance and Safety of Crops & Foods 2011 3 104 122 10.1111/j.1757-837X.2011.00104.x20832442

[B11] Iversen C Lancashire L Waddington M Forsythe S Ball G Identification of *Enterobacter sakazakii *from closely related species: The use of Artificial Neural Networks in the analysis of biochemical and 16S rDNA data BMC Microbiology 2006 6 28 10.1186/1471-2180-6-28 PMC1421405 16533390

[B12] Baldwin A Loughlin M Caubilla-Barron J Kucerova E Manning G Dowson C Forsythe S Multilocus sequence typing of *Cronobacter sakazakii *and *Cronobacter malonaticus *reveals stable clonal structures with clinical significance which do not correlate with biotypes BMC Microbiology 2009 9 1 223 10.1186/1471-2180-9-223 19852808 PMC2770063

[B13] *Cronobacter *Multi Locus Sequence Typing Database http://pubmlst.org/cronobacter Date of last accession 16/03/2011

[B14] Joseph S Forsythe SJ Predominance of *Cronobacter sakazakii *ST4 in neonatal infections Emerg Infect Dis 2011 17 1713 1715 10.3201/eid1709.110260 21888801 PMC3322087

[B15] Kucerova E Clifton SW Xia XQ Long F Porwollik S Fulton L Fronick C Minx P Kyung K Warren W Fulton R Feng D Wollam A Shah N Bhonagiri V Nash WE Hallsworth-Pepin K Wilson RK McClelland M Forsythe SJ Genome sequence of *Cronobacter sakazakii *BAA-894 and Comparative Genomic Hybridization Analysis with other *Cronobacter *species PLOS One 2010 5 3 e9556 10.1371/journal.pone.0009556 20221447 PMC2833190

[B16] Kanehisa M Goto S Kawashima S Nakaya A The KEGG databases at GenomeNet Nucleic Acids Res 2002 30 1 42 10.1093/nar/30.1.42 11752249 PMC99091

[B17] Kothary MH McCardell BA Frazar CD Deer D Tall BD Characterization of the zinc- containing metalloprotease encoded by *zpx *and development of a species-specific detection method for *Enterobacter sakazakii* Appl and Env Microbiol 2007 73 13 4142 4151 10.1128/AEM.02729-06 PMC1932767 17483271

[B18] Stephan R Lehner A Tischler P Rattei T Complete genome sequence of *Cronobacter turicensis *LMG 23827, a food-borne pathogen causing deaths in neonates J Bacteriol 2011 193 1 309 10.1128/JB.01162-10 21037008 PMC3019923

[B19] Iversen C Forsythe SJ Isolation of *Enterobacter sakazakii *and other Enterobacteriaceae from powdered infant formula milk and related products Food Microbiol 2004 21 771 776 10.1016/j.fm.2004.01.009

[B20] Muytjens HL Roelofs-Willemse H Jaspar GHJ Quality of powdered substitutes for breast milk with regard to members of the family *Enterobacteriaceae* J Clin Microbiol 1988 26 4 743 746 3284901 10.1128/jcm.26.4.743-746.1988 PMC266435

[B21] Muytjens HL Zanen HC Sonderkamp HJ Kollée LA Washsmuth K Farmer JJ Analysis of eight cases of neonatal meningitis and sepsis due to *Enterobacter sakazakii* J Clin Microbiol 1983 18 115 120 6885983 10.1128/jcm.18.1.115-120.1983 PMC270753

[B22] Himelright I Harris E Lorch V Anderson M *Enterobacter sakazakii *infections associated with the use of powdered infant formula -Tennessee JAMA 2001 287 2204 2205 11987295

[B23] Caubilla-Barron J Townsend S Cheetham P Loc-Carrillo C Fayet O Prere MF Forsythe SJ Genotypic and phenotypic analysis of *Enterobacter sakazakii *strains from an outbreak resulting in fatalities in a neonatal intensive care unit in France J Clin Microbiol 2007 45 3979 3985 10.1128/JCM.01075-07 17928419 PMC2168550

[B24] Townsend S Hurrell E Forsythe S Virulence studies of *Enterobacter sakazakii *isolates associated with a neonatal intensive care unit outbreak BMC Microbiol 2008 8 64 10.1186/1471-2180-8-64 PMC2386127 18423002

[B25] Hurrell E Kucerova E Loughlin M Caubilla-Barron J Hilton A Armstrong R Smith C Grant J Shoo S Forsythe S Neonatal enteral feeding tubes as loci for colonisation by members of the *Enterobacteriaceae* BMC Infect Dis 2009 9 146 10.1186/1471-2334-9-146 PMC2749046 19723318

[B26] Pagotto FJ Nazarowec-White M Bidawid S Farber JM *Enterobacter sakazakii*: infectivity and enterotoxin production *in vitro *and *in vivo* J Food Protect 2003 66 370 377 10.4315/0362-028x-66.3.370 12636287

[B27] Aldová E Hausne O Postupa R Tween esterase activity in *Enterobacter sakazakii* Zentralblatt fuer Bakteriologie Mikrobiologie und Hygiene Series A 1983 256 103 108 6659742

[B28] Iversen C Waddington M Farmer JJ III Forsythe S The biochemical differentiation of *Enterobacter sakazakii *genotypes BMC microbiol 2006 6 94 10.1186/1471-2180-6-94 PMC1634753 17067387

[B29] Smith P Tomfohrde K Rhoden D Balows A API system: a multitube micromethod for identification of *Enterobacteriaceae* Appl Microbiol 1972 24 3 449 4562482 10.1128/am.24.3.449-452.1972 PMC376540

[B30] O'Hara CM Miller JM Evaluation of the ID 32E for the identification of Gram-negative glucose-fermenting and glucose-non-fermenting bacilli Clinical Microbiology and Infection 1999 5 5 277 281 10.1111/j.1469-0691.1999.tb00141.x 11856267

[B31] Humble M King A Phillips I API ZYM: a simple rapid system for the detection of bacterial enzymes J Clin Pathol 1977 30 3 275 10.1136/jcp.30.3.275 845275 PMC476372

[B32] Dempster AP Laird NM Rubin DB Maximum likelihood from incomplete data via the EM algorithm Journal of the Royal Statistical Society Series B (Methodological) 1977 39 1 1 38

[B33] Witten IH Frank E Data Mining: Practical machine learning tools and techniques 2005 Morgan Kaufmann Pub

